# The Difficult-to-Treat del 17 p Patient—A Case Report in Chronic Lymphocytic Leukemia

**DOI:** 10.3390/medicina58010033

**Published:** 2021-12-24

**Authors:** Ana-Maria Moldovianu, Ana Manuela Crisan, Zsofia Varady, Daniel Coriu

**Affiliations:** 1 Department of Hematology and Bone Marrow Transplant, Fundeni Clinical Institute, 258 Fundeni Street, 022328 Bucharest, Romania; ana.moldovianu@gmail.com (A.-M.M.); varadyzsofia@gmail.com (Z.V.); daniel_coriu@yahoo.com (D.C.); 2 Department of Hematology, University of Medicine and Pharmacy “Carol Davila”, 8 Eroii Sanitari Blvd, 050474 Bucharest, Romania

**Keywords:** chronic lymphocytic leukemia, 17 p deletion, allogeneic stem cell transplantation

## Abstract

Chronic lymphocytic leukemia (CLL) treatment strategies have evolved to include mechanism-driven drugs but now raise new questions regarding their optimum timing and sequencing. In high-risk patients, switching from pathway inhibitors to allogeneic stem cell transplantation (allo-HCT) is still a matter of intense debate. We report the case of a CLL patient with 17 p deletion treated with ibrutinib as a bridge to allo-HCT. Early relapse after allo-HCT urged the initiation of salvage therapy, including donor lymphocytes infusions, ibrutinib, and venetoclax. We aim to outline and discuss the potential benefits of novel therapies, the current role of allo-HCT in CLL, drug timing and sequencing, and the unmet need to improve the long-term outcome of high-risk CLL patients.

## 1. Introduction

Chronic lymphocytic leukemia (CLL) accounts for 30% of all leukemias in adults and is the commonest type in Western countries [1]. It is an indolent lymphoproliferative disorder exhibiting a broad spectrum of clinical, biological, and genetic features, with a life expectancy of 2 to 20 years. Timely identification and treatment of patients with a high risk for progression have fueled the latest research. State-of-the-art algorithms incorporating adaptive risk strategies with biologically and genetically based prognostic enrichment improved outcomes significantly. However, the long-term prognosis remains poor for CLL patients with 17 p deletion (del 17 p) [2].

Targeted drugs, including B-cell receptor (BCR) inhibitors (e.g., ibrutinib, acalabrutinib, zanubrutinib, idelalisib, and duvelisib) or B-cell lymphoma 2 (BCL2) antagonists (e.g., venetoclax), marked a paradigm shift in CLL treatment. The overall response rate (ORR) of ibrutinib, the inaugural Bruton Tyrosine Kinase (BTK) inhibitor, in relapsed/refractory (R/R) settings was nearly 90%, with a median progression-free survival (PFS) of 44 months [3]. In contrast, ibrutinib was less efficacious in del 17 p patients but still superior to chemo-immunotherapy, showed an ORR of 83% after a median follow-up of 28 months, and resulted in a 24-month PFS and overall survival (OS) rates of 63% and 75% [4]. Venetoclax produced a high ORR when used alone or in combination with rituximab in R/R CLL [5], achieving in del 17 p patients an ORR of 77%, with an estimated 24-month PFS and OS of 54% and 73%, respectively [6]. Acalabrutinib, a second-generation BTK inhibitor, produced an ORR of 93% in del 17 p R/R patients [7]. The new next-generation BTK inhibitor, zanubrutinib, was recently investigated as a frontline monotherapy in CLL patients with del 17 p. It showed a robust ORR of 94.5% after 18.2 month-median follow-up, with a duration of response (DOR) greater than 12 months in 92.8% of patients [8].

On the other hand, although phosphatidylinositol 3-kinase (PI3K) inhibitors (i.e., idelalisib and duvelisib) performed well in R/R CLL, including high-risk patients [9,10], they have remained underused due to their weak safety profiles.

Traditionally, allogeneic stem cell transplant (allo-HCT) was the treatment choice for all high-risk transplant eligible CLL patients in R/R settings [11] and has remained the only curative therapy. Nevertheless, given the very effective novel targeted drugs, its role needs revision.

## 2. Case Report

In May 2014, 2 years after diagnosis, a 40-year-old female patient with CLL was admitted with relapsed disease following initial immuno-chemotherapy with R-CHOP (i.e., rituximab, cyclophosphamide, doxorubicin, vincristine, and prednisone) and FCR (i.e., fludarabine, cyclophosphamide, and rituximab) that ended in November 2013. She had marked lymphadenopathy, splenomegaly, severe anemia, hyperleukocytosis with lymphocytosis, and mild thrombocytopenia. Peripheral blood flow cytometry analysis was consistent with CLL, bone marrow studies showed massive and diffuse infiltration with clonal small B lymphocytes, and fluorescence in situ hybridization (FISH) detected del 17 p in 38% of nuclei, indicating a high risk. Tissue type analysis of her siblings found a 10/10 HLA matched related donor.

Initially, she had a prompt and side-effect-free response to ibrutinib treatment, achieving partial remission 12 months later with residual disease limited to bone marrow (i.e., 25% clonal B lymphocytes on bone marrow immunohistochemistry analysis).

The BTK inhibitor was stopped in January 2016 and followed by an uneventful allo-HCT with reduced-intensity conditioning (RIC), resulting in 1- and 3-month chimerism of 100% and 83% of donor cells. The RIC regimen consisted of fludarabine 150 mg/m^2^ total dose, melphalan 140 mg/m^2^ total dose, and cyclosporine and mycophenolate mofetil as graft-versus-host disease (GVHD) prophylaxis.

Despite immunomodulation-based interventions to boost the graft-versus-leukemia (GVL) effect, the response was short-lived, as lymphadenopathy and thrombocytopenia ensued 5 months later, and chimerism at 6 months dropped to 56% of donor cells. Consequently, donor lymphocyte infusions (DLIs) were delivered as follows: 1st dose, 1 × 10^6^ CD3/kg/body weight (BW) at 6 months; 2nd dose, 1.18 × 10^6^ CD3/kg/BW at 7 months; 3rd dose, 0.9 × 10^6^ CD3/kg/BW at 8 months; 4th dose, 1 × 10^6^ CD3/kg/BW at 9 months; 5th dose, 1 × 10^7^ CD3/kg/BW at 10 months; and 6th dose, 600 × 10^7^ CD3/kg/BW at 11 months post-allo-HCT. Concomitantly, ibrutinib was started and achieved another partial remission, followed by disease progression 2 years later. Notably, given the bulky (>5 cm) lymphadenopathy, metabolic imaging with 18-fluorine fluorodeoxyglucose positron emission tomography-computed tomography (18F-FDG PET/CT) refuted the case of Richter’s transformation.

**Figure 1:**
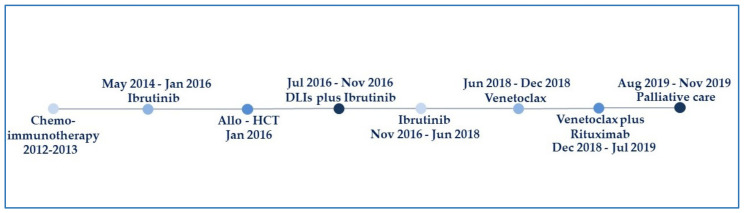
Treatment sequence (see text for further details). Allo-HCT, allogeneic stem cell transplant; DLIs, donor lymphocyte infusions.

Venetoclax was initiated in 2018, but untoward effects were prohibitive (i.e., grade 4 neutropenia and thrombocytopenia) and forced half-dosing and, later, combination therapy with rituximab. Later, the course was protracted and paved with CLL pulmonary involvement confirmed by CT imaging with negative viral, fungal, and bacterial screening. Pancytopenia and recurrent infections soon ensued. In 2019, gemcitabine-based chemotherapy proved futile and was replaced by a palliative care policy a few months before demise (Figure 1).

## 3. Discussion

Heightened risk and poor prognosis stem from two features, an early relapse (i.e., less than 2 years) after purine-analog-based therapy and del 17 p. Notably, the latter was only determined 2 years after the initial diagnosis and following extensive immune-chemotherapy. This delay is highly consequential, as del 17 p can be intrinsically linked to CLL and advise against traditional chemotherapy, or chemotherapy itself may elicit del 17 p [12]. Indeed, the reported incidence of high-risk genetics is close to 10% at diagnosis yet increases to up to 50% with relapse [2,13]. A short-lived remission after FCR suggests that del 17 p may have been present from the very beginning in our patient. Timely confirmation of del 17 p is paramount, as it evades unnecessary and potentially harmful treatments (e.g., FCR) and hastens targeted therapy initiation (e.g., ibrutinib and venetoclax). Beginning with the first hierarchical OS prognostic model based on four-probe FISH analysis (i.e., del 17 p, 11 q deletion, 13 q deletion, and trisomy 12), many studies established that del 17 p provides the worst outcome, with a median OS of less than 3 years [2,14,15].

Del 17 p CLL exhibits marked diversity in clinical behavior, suggesting that cytogenetic and genomic anomalies other than del 17 p may influence the outcome. The percentage of cells with del 17 p has prognostic significance, with a small clone size signaling a favorable outcome, but cut-offs are yet to be established [16,17,18].

The deletion 17 p results in the loss of one TP53 copy, and in 81% of cases is associated with somatic mutations in the second allele, causing biallelic TP53 gene inactivation and a dismal outcome [19]. Clonal mutations carry an even worse prognosis [19]. Subclonal TP53 mutations in del 17 p patients provide a slightly better OS, and monoallelic TP53 disruption has an intermediate OS between wild-type TP53 and biallelic TP53 aberrations [19]. Additionally, a complex karyotype (CK) (i.e., three or more chromosomal aberrations) identified by chromosome band analysis further aggravates the clinical outcome of del 17 p patients and shortens the OS time [20,21,22]. Recently, a study proposed a new hierarchical OS model that only places high-CK (i.e., five or more anomalies) patients at increased risk independently of TP53 abnormalities [22].

To date, allo-HCT constitutes the only treatment option with curative potential [11]. CLL is highly sensitive to GVL alloimmune effects, resulting in long-term remissions after non-myeloablative or RIC allo-HCT [23,24,25,26,27]. Furthermore, the RIC allo-HCT procedure may counteract del 17 p or FCR resistance [11,23,27]. Given an initial lack of response with standard chemotherapy, a non-cytotoxic drug was used as a bridge to transplantation. Following partial remission with ibrutinib monotherapy, two options emerged. The first was to reinforce remission with allo-HCT, and the second was to postpone the allo-HCT and continue the BTK inhibitor until disease progression or toxicity, whichever came up first. For several reasons, the transplant was prioritized, including the scant high-quality data comparing allo-HCT and novel drugs, a poor starting prognosis, the young age, and the availability of a highly compatible donor. Despite favorable initial results, relapse soon ensued with chimerism halving at 6 months.

Our case is in line with Dreger P. et al., who reported an increased incidence (30%) of early relapse after allo-HCT in ibrutinib pre-exposed CLL patients [28] compared with ibrutinib-naïve patients (16%) [29]. The underlying mechanisms of these observations are yet to be determined, but several insights can be garnered from current data. Firstly, the depth and durability of response before allo-HCT may be lower after ibrutinib than after chemo-immunotherapy [30]. Secondly, ibrutinib exhibits immunomodulatory effects that curb the GVL [31] and the GVHD [32]. Lastly, a shorter drug exposure before transplantation and ibrutinib resistance constitute alternative explanations for the authors’ findings. The pragmatic message to pick out from the above is that the optimum timing to deploy allo-HCT immediately follows the best possible ibrutinib response.

Before the emergence of novel targeted drugs, studies showed that, for poor-risk CLL, allo-HCT could result in long-term minimal residual disease (MRD)-negative survival in up to one-half of the patients irrespective of genomic risk profile [23].

Despite long-term disease control and low early-death rate, the allo-HCT-related OS (62% at 2 years, 35% at 10 years) and event-free survival (49% at 2 years, 28% at 10 years) decrease over time, whereas non-relapse mortality increases to 40% at 10 years [33]. Additionally, on long-term follow-up, novel targeted drugs ensure high response rates and sustained tolerability in treatment-naive and R/R CLL patients [34,35]. Allo-HCT should be weighed against novel therapies, considering comorbidities, high-risk features, side-effect profiles, response rates and duration. A risk-benefit assessment is always mandatory. As a case in point, the allo-HCT may constitute a feasible treatment option in a young patient with R/R CLL, low comorbidity score, high-risk disease features, and well-matched donor. Furthermore, the optimum timing for allo-HCT is yet another topic of intense debate. The novel targeted drugs are currently the preferred first- and second-line therapy in most cases. By contrast, allo-HCT is downgraded and requires well-controlled disease before inclusion.

Treatment after the failure of allo-HCT remains unstandardized. In our case, ibrutinib was resumed and combined with a 6-month course of DLIs since GVHD was absent. Evidence supporting the use of DLIs within this context is merely anecdotal. For example, one study involving 28 patients with low-grade lymphoid malignancies found 76.5% and 91.6% cumulative response rates after DLIs-based treatment of disease progression and persistent mixed chimerism [36]. Other authors reported a potential benefit of pre-emptive DLIs in early relapse with detectable MRD and mixed chimerism [37]. However, the chimerism fall-off progressed, and ibrutinib was probably responsible for a second yet temporary disease control. Indeed, several studies explored ibrutinib to treat relapse after allo-HCT and reported similar efficacy and safety profile with non-transplant high-risk settings [38,39]. Ibrutinib was well tolerated and triggered no GVHD manifestations in our case, reinforcing the belief that BTK inhibitors and allo-HCT are complementary [40] and not mutually exclusive treatments for high-risk CLL patients.

Venetoclax, alone or in combination with rituximab, marked the second stage of salvage therapy after ibrutinib failure. Symptom alleviation was rapid but high-grade pancytopenia emerged consistent with published data [41]. This forced an irregular schedule and dosing, causing inadequate blood concentrations. The granulocyte colony-stimulating factor (GCSF) helps maintain venetoclax dose intensity, but thrombocytopenia correction remains a daunting task at the bedside.

Long therapy exposure risks secondary resistance to novel drugs (i.e., BCR inhibitors and BCL-2 antagonist) [42,43], hence the time-limited therapy rationale. Venetoclax-based therapy favors fixed-duration strategies based on increased CR rates (53%) and undetectable MRD (uMRD) (61%) achievement [44]. Additionally, uMRD benefited the 5-year PFS, OS, and DOR (i.e., 86%, 56%, and 58%, respectively) [44] and was associated with the best outcome. The PFS rates at 18 months were 90.3% in patients with uMRD (i.e., <1 CLL cell/10,000 leukocytes), 64.4% in patients with low MRD (i.e., 10^−4^ to 10^−2^) positivity, and 8.3% in patients with high MRD positivity (<10^−2^) [35]. Nevertheless, lower rates of uMRD were achieved in del 17 p patients, and venetoclax produced an estimated median DOR of 33.2 months, with an estimated 24-month PFS and OS of 54% and 73% [6].

Experimental treatment options may have included chimeric antigen receptor T (CAR-T) cells, reversible BTK inhibitors (i.e., Loxo-305), and neoteric multidrug combinations (e.g., ublituximab and ibrutinib; ibrutinib, venetoclax, and obinutuzumab).

## 4. Conclusions

Disease progression and relapse have remained largely unmanageable in high-risk CLL patients, with del 17 p mutation particularly carrying a dismal prognosis regardless of treatment. Future research must address several critical issues to improve outcomes, including optimum timing and sequencing of allo-HCT and the various available small molecules.

## Data Availability

Not applicable.
